# Comparison of Phytochemicals, Antioxidant and Anti-Inflammatory Properties of Sun-, Oven- and Freeze-Dried Ginger Extracts

**DOI:** 10.3390/foods8100456

**Published:** 2019-10-06

**Authors:** Iswaibah Mustafa, Nyuk Ling Chin, Sharida Fakurazi, Arulselvan Palanisamy

**Affiliations:** 1 Department of Process and Food Engineering, Faculty of Engineering, Universiti Putra Malaysia, 43400 UPM Serdang, Selangor, Malaysia; is.greenish@gmail.com; 2 Laboratory of Vaccines and Immunotherapeutics, Institute of Bioscience, Universiti Putra Malaysia, 43400 UPM Serdang, Selangor, Malaysia; sharida@upm.edu.my (S.F.); arulbio@gmail.com (A.P.)

**Keywords:** *Halia Bara*, functional food, ginger extract, nitric oxide, RAW 264.7 macrophages

## Abstract

The effects of different drying methods, including sun-, oven-, and freeze-drying on the changes in the antioxidant and anti-inflammatory activities of ginger (*Zingiber officinale* var. Rubra) rhizome were studied. Sun-, oven-, and freeze-dried ginger showed a significant (*p* < 0.05) increase in phenolic content by 1.79, 1.53, and 1.91-fold; flavonoid content increased by 6.06, 5.27, and 4.90-fold; FRAP increased by 3.95, 3.51, and 3.15-fold; ABTS^•+^ scavenging activity increased by 2.07, 1.72, and 1.61-fold; and DPPH^•^ inhibition increased by 78%, 58%, and 56%, respectively. Dried ginger also exhibited better inhibitory effects on the lipopolysaccharides-induced nitric oxide production in murine macrophage RAW 264.7. The drying process demonstrated a positive effect on the bioactivities of ginger. The sun-dried ginger exhibited the most potent antioxidant properties with the best enhanced anti-inflammatory activity followed by the oven-dried ginger and lastly, the freeze-dried ginger.

## 1. Introduction

Ginger is the rhizome of the flowering plant, *Zingiber officinale*, a perennial herb from the family *Zingiberaceae* that is widely used as food flavoring and also as a natural source of functional foods and nutraceuticals. Traditionally, ginger is used as remedy in folk medicine to treat a wide range of ailments, including nausea and vomiting, colds and flu symptoms, migraine and headaches, menstrual periods, as well as muscular and rheumatic disorders [1,2]. The health-promoting behavior of ginger is attributed to its rich and potent antioxidant phytochemicals. These phytochemicals have many health benefits and contribute to the prevention of major oxidation-linked disease, such as cancer promotion, arthritis, cognitive diseases, diabetes mellitus, and cardiovascular diseases, etc. [3]. 

In recent years, many reviews on ginger have reported its beneficial bioactivities such as its antioxidant properties and anti-inflammatory action in biological systems [1,2,4,5]. Ginger has been reported to protect humans from oxidative stress and inflammation related disorders [4]. Oxidative stress is related to an imbalance between the production of free radicals and the antioxidant defense system. Free radicals and their uncontrolled production initiate and propagate the oxidation chain reactions and lead to inflammation development [6]. In inflammatory disorders, nitric oxide (NO) is secreted excessively and overproduction of NO or prolonged inflammation result in the development of disease. In the antioxidant defense system, enzymes (made in the body) and essential nutrients (found in foods) deactivate these free radicals and help to decrease their harmful effects. The defense system also turns the free radicals into waste products that can be excreted by the body. Ginger, a common herb-based functional food, has been reported for its potential protective benefits against oxidation and inflammation related disorders [4,7].

Fresh ginger, has a high moisture content of 78.89% (United States Department of Agriculture Nutrient Database Release 28) [8], and is classified as a highly perishable commodity. Ginger with high moisture content is highly susceptible to spoilage through micro-organism growth and moisture-mediated deteriorative reactions. Thus, drying ginger is a good post-harvest process that extends its shelf life, whilst preserving its natural phytochemicals and enhancing its bioactivities [9]. Sun-, oven- and freeze-drying are the most common drying methods for food materials, and these methods have their own particular characteristics. When water content is reduced to a low level, growth of micro-organisms, enzymatic reactions and other detrimental changes are inhibited [9]. Oven-drying is often used because it lowers investment and operating costs, but the high temperature applied results in inferior product quality [10]. Conversely, freeze-drying can help to maintain the product’s quality attributes, such as nutrients, color, flavor with indistinguishable changes to the original product, but it involves high production costs [11]. The sun-drying method has the advantage simplicity and it is an economically affordable process. In addition, it has been recently hypothesized that sun-drying, due to ultraviolet-B (UVB) rays may add the extra effect of vitamin D to the dried samples [12]. The UVB from sunlight has also been reported to induce changes in the accumulation of bioactive phytochemicals such as phenols and flavonoids, thus promoting better biological activities for human health [13]. Open sun-drying of ginger [14,15] has been widely practiced in many urban and rural areas of hot climate countries, probably because it is a cheaper method of drying even though it has likely been used without the knowledge of the possible additional benefits of the sun rays. Jayashree et al. [15] have reported that dried ginger obtained by sun-drying retained the maximum essential oil and oleoresin content compared to those obtained by solar and convective air drying. 

In Malaysia, ginger (*Zingiber officinale*) var. Rubra, known as *Halia Bara* is the most highly sought and expensive ginger type used as a major herb, health food and in traditional medicine by the locals. Due to its nutritive value and the presence of potent bioactive compounds, the use of this ginger rhizome has increased drastically, resulting in an increase of 100% in market demand in the last 5 years (Food and Agriculture Organization) [16]. However, most of the ginger is used in its fresh form. Hence, the present study investigated the effects of different drying methods on its phenolic and flavonoid contents, reductive ability (FRAP), radical scavenging activity (ABTS^•+^ and DPPH^•^) and anti-inflammatory behavior through the NO-inhibitory activity. Other studies on drying of similar food products, such as Lamiaceae herbs [17] and green tea [18] have reported an increase in phenolic contents and antioxidant properties. The aim of this study was to compare the basic sun-drying method with equipment-based drying methods and to reveal its advantages.

## 2. Materials and Methods 

### 2.1. Sample Preparation

Fresh rhizome of *Halia Bara* grown in the farm area of Tendong, Pasir Mas, Kelantan, Malaysia was obtained during the period of December 2014 to January 2015. The matured gingers (at 8 months) were sliced into thicknesses of less than 5mm and subjected to sun-, oven-, and freeze-drying. Fresh ginger was used as control. 

### 2.2. Drying Treatment and Sample Processing

Freeze-dried ginger was prepared by freezing at −30 °C in a freezer (Haier, Biomedical, Malaysia), and then lyophilized in a freeze-dryer (Coolsafe Benchtop, Scanvac, Sweeden) for 3 days. For oven-drying, ginger slices were spread on stainless steel trays (size 30 cm × 40 cm) and dried in an oven (UM500, Memmert GmbH, Schwabach, Germany) at 60 °C for 4 days. For sun-drying, ginger slices were spread out on a 70 cm diameter round rattan tray and dried under direct sunlight at temperatures between 28 and 44 °C, for 3 days with about 36 h of daylight. Mid-day temperature reached up to 44 °C. The completely dried samples, which had 7–10% moisture content, were pulverized separately using an electrical food blender (RT-02A, Taiwan). The powdered samples were packed in air-tight containers and stored at 4 °C (Model SD-700, Protech, Malaysia) for extraction studies. Fresh samples of ginger were crushed for subsequent analysis.

### 2.3. Extraction Procedures

Ginger samples of 10 g (fresh and dried) were extracted by ethanol at the ratio of 1:10 (10:100 mL) in conical flasks. The flasks were shaken at 150 rpm to mix the samples with solvent in an incubator (Wisecube WIS-30, Daihan Scientific, Korea) for 24 h at room temperature. After standing overnight, the extracts were filtered through filter paper (Fioroni Grade 601) to separate the liquid. The residue mass was then re-extracted with the same solvent to ensure complete extraction. The collected extract was combined and evaporated to dryness at 40 °C to yield solid extract. The solid extract was weighed to determine the yield of the soluble constituents using Equation (1):

(1) $$\mathrm{Extract}\;\mathrm{Yield}\%=\frac{\mathrm{Weight}\;\mathrm{of}\;\mathrm{solid}\;\mathrm{extract}}{\mathrm{Weight}\;\mathrm{taken}\;\mathrm{for}\;\mathrm{extraction}\;}\;\times \;100\%.$$

Five mg of solid extract was used for the antioxidant and anti-inflammatory activities analyses where 1 mg/mL standard stock solutions in ethanol solvent were prepared and stored at 4 °C. 

### 2.4. Determination of Phenolic Content

The assay was conducted following the Folin-Ciocalteau method [19]. Aliquots (100 µL) of ethanol extracts were made up to 1 mL with distilled water. Then, 0.5 mL Folin-Ciocalteu reagent (diluted 1:1 with water, *v*/*v*) and 20% (*w*/*v*) sodium carbonate (2.5 mL) was added. The mixture was left at room temperature for 40 min in the dark. Absorbance of the resulting blue color was measured at 725 nm using a spectrophotometer (Ultrospec 3100 pro, Amersham Biosciences, Piscataway, NJ, USA). The results were expressed as milligram of gallic acid equivalents (GAE) per gram of dry extract. 

### 2.5. Determination of Flavonoid Content

Flavonoid content was determined following the method described by Zhishen et al. [20]. Extract solution (100 µL) was made up to 2 mL with distilled water. Then, 0.15 mL sodium nitrite (5%, *w*/*v*) was added to the mixture. After 6 min incubation, 0.15 mL aluminium chloride (10%, *w*/*v*) was added to the mixture. The mixture was allowed to stand for 6 min before 2 mL sodium hydroxide (4%, *w*/*v*) was added and the total volume of the mixture was made up to 5 mL with distilled water. The solution was vigorously mixed and allowed to stand in the dark for 15 min. Absorbance of the resulting yellowish-orange color was measured at 510 nm. The results were expressed as milligram of rutin equivalents (RE) per gram of dry extract. 

### 2.6. Assessment of Antioxidant Activity

#### 2.6.1. Ferric-Reducing Antioxidant Power (FRAP) 

The antioxidant potential of ginger extract was determined using the FRAP assay described by Pulido et al. [21]. A potential antioxidant reduces ferric ion (Fe^3+^) to the ferrous ion (Fe^2+^). The ferric ion reagent consists of 20 mmol/L TPTZ (2,4,6-tripyridyl-s-triazine) in 40 mmol/L HCl, 20 mmol/L FeCl_3_·6H_2_O and 300 mmol/L acetate buffer pH 3.6 in the ratio of 1:1:10. This 900 µL of FRAP reagent was added to the 30 µL of sample extract and the mixture was made up to 1 mL with distilled water. The mixture solution was vigorously shaken and the absorbance was measured at 593 nm after 30 min of incubation. Methanol solutions of FeSO_4_⋅7H_2_O were prepared as standard curve between 10 to 100 µmol/L. Results were expressed as mmol Fe(II) equivalents per gram of dry extract. 

#### 2.6.2. Radical Scavenging Activity on ABTS Radical Cation

The radical cation 2,2′-azino-bis(3-ethylbenzothiazoline-6-sulfonic acid) (ABTS^•+^) assay was based on the method of Re et al. [22]. The ABTS reagent was freshly prepared by mixing an oxidant (2.45 mmol/L potassium persulfate) and 7 mmol/L ABTS stock solution and was incubated in the dark for 12–16 h at 37 °C. The reaction produced a stable, dark blue-green radical solution. The solution then was diluted with ethanol to an absorbance of 0.70 (±0.02) at 734 nm. Diluted ABTS^•+^ solution (0.9 mL) was added to the 100 µL of extract sample and mixed uniformly and absorbance was measured at 734 nm after 30 min incubation. Trolox standards (final concentration 0–15 µmol/L) were used as an antioxidant standard. Results were expressed as mmol of Trolox equivalents antioxidant capacity (TEAC) per gram of dry extract.

#### 2.6.3. Radical Scavenging Activity on DPPH Radical

The stable radical 1,1-diphenyl-2-picryl-hydrazyl (DPPH^•^) scavenging activity was determined using the method proposed by Sowndhararajan et al. [23] by preparing DPPH solution (0.1 mmol/L) in absolute ethanol. Ginger extracts at various concentrations was taken and the volume was adjusted to 50 µL with methanol. Then, 950 µL of DPPH solution was added and allowed to react for 20 min in a dark place. Absorbance of the sample was measured at 517 nm. IC_50_ values were determined by plotting a graph of the percentage of inhibition against the concentration using linear regression analysis. IC_50_ values represent the concentration needed to scavenge 50% of free radicals in a sample. Antioxidants with higher scavenging abilities have lower IC_50_ values.

### 2.7. Assessment of Anti-inflammatory Activity

#### 2.7.1. Cell Culture Experiment

The murine macrophage cell line RAW 264.7 were purchased from American Type Culture Collection (Manassas, VA, USA) and cultured in Dulbecco’s modified Eagle’s medium (DMEM) supplemented with 10% fetal bovine serum (FBS), 1% penicillin-streptomycin (P/S) at 37 °C in a 5% CO_2_ incubator. The tested samples were diluted with culture medium into different concentrations. Cell viability was then evaluated using the 3-(4,5-dimethylthiazol-2-yl)-2,5-diphenyltetrazolium bromide (MTT) assay [24]. MTT assay was performed to examine cell viability to ensure that the ginger extracts exhibited no cytotoxicity against RAW 264.7 cells at their effective concentrations.

#### 2.7.2. Determination of Secreted NO Amounts

The concentration of NO in the medium was then determined by the Griess reaction using the protocol reported in [24]. The amount of nitrite released, essentially the primary stable breakdown of NO in the media, was calculated from the sodium nitrite (NaNO_2_) standard curve.

#### 2.7.3. Inhibitory Effect on Lipopolysaccharide (LPS)-Induced Nitric Oxide (NO) Production 

The capacity (%) of each ginger extract to inhibit the production of the inflammatory mediator (NO) was calculated as follow:

(2) $$\mathrm{NO}\;\mathrm{inhibition}\left(\%\right)=\;\frac{1-\left(C_{\mathrm{ginger}+\mathrm{LPS}}-{\;C}_{-\mathrm{LPS}}\right)}{C_{+\mathrm{LPS}}\;-{\;C}_{-\mathrm{LPS}}}\;\times \;100\%,$$

where C_ginger+LPS_ is the inflammatory mediator concentration (NO) of conditioned medium collected from cell culture co-treated with ginger extract and 1 µg/mL of LPS. C_-LPS_ is the concentration of conditioned medium collected from cell culture only treated with DMEM media. C_+LPS_ is the concentration of conditioned medium collected from cell culture treated with LPS (1 µg/mL) without any other intervention component.

### 2.8. Statistical Analysis 

Statistical analysis of the data was conducted using Minitab 16 statistical analysis software (Minitab Inc., State College, PA, USA). Results were expressed as mean ± standard deviations (SD). Differences among drying methods were tested by one way-ANOVA followed by Tukey’s test for mean comparisons. *p* values less than 0.05 were considered significant. 

## 3. Results and Discussion

### 3.1. Phenolic and Flavonoid Contents of Ginger Extracts 

Table 1 shows the extract yield and phenolic and flavonoid content of fresh and dried gingers prepared using different drying methods. The extractive value of dried ginger, which ranged from 8.21 to 9.87% is greater than that of the fresh ginger at 3.40%, and the highest value was from the sun-dried ginger. According to Hossain et al. (2010), the drying process makes the tissue samples more brittle, which in turn results in the breakdown of cell wall during milling, thus promoting the homogenization steps in the extraction process. During the extraction, the broken cells liberate more extract compounds into the solvents when they are shaken overnight. Shirsath et al. [25] have reported that dried samples are also higher in porosity when the diffusion rate of solute and solvent extraction are enhanced and result in higher extract yields than fresh sample.

The phenolic content, expressed as GAE, of sun-, oven-, and freeze-dried extracts of ginger is significantly (*p* < 0.05) higher than fresh extract by 1.79, 1.53, and 1.91-fold, respectively, with freeze-dried extracts being the highest. The high level of phenolic content in freeze-dried samples might be due to the formation of ice crystals within the plant matrix during freezing, which may cause greater disruption of the cell wall structure, allowing for accelerated liberation of cellular components and accessibility of the solvent [26]. The flavonoid content of dried ginger extracts was also increased by the sun-, oven-, and freeze-drying. The values increased significantly (*p* < 0.05) by 6.06, 5.27, and 4.90-fold, respectively. Phenols and flavonoids are natural polyphenolics that may render their effect via anti-oxidative action in biological systems, acting as scavengers of singlet oxygen, removing free radicals, activating antioxidant enzymes and inhibiting oxidases [3].

### 3.2. Antioxidant Activity of Ginger Extracts

In FRAP assay, the Fe (III) reduction is often used as a measurement of electron donating activity, which is a significant reflection of the antioxidant activity. In an antioxidative action, reducing agents break free radical chains by donating a hydrogen atom to stabilize it [27]. Table 2 shows that the antioxidant activities of reducing power (FRAP) improved in dried ginger. The reductive ability of sun-, oven-, and freeze-dried ginger extracts increased significantly by 3.95, 3.51, and 3.15-fold, respectively, when compared with the fresh extract. In accordance to our results, sun-dried extracts might contain higher amounts of reductone, which could react with free radicals to stabilize and break the oxidation chain [28].

The antioxidant activity of ginger with regards to their ability to scavenge free radicals was also determined. The formation of free radicals and other reactive oxygen species (ROS) is one of the main reasons for the occurrence of degenerative diseases, including inflammation and others. Table 2 shows the improvement in the ability of ginger extracts to scavenge ABTS radical cation and DPPH free radical in dried samples. The radical scavenging activity on ABTS radical cation also increased in the same manner as FRAP, where sun-, oven-, and freeze-drying respectively produced a 2.07, 1.72, and 1.61-fold increase. For DPPH radical, dried ginger extracts exhibited lower IC_50_, which indicates they have better ability to scavenge DPPH^•^. The DPPH^•^ inhibition of dried extracts increased by 78%, 58%, and 56%, respectively. The capacity of ginger extracts to scavenge ABTS^•+^ and DPPH^•^ could be related to the nature of ginger polyphenols and their hydrogen donor molecules, which act as potent antioxidants [23].

Sun-dried ginger possessed the highest antioxidant activity as reflected in its FRAP, ABTS^•+^ scavenging activity and DPPH^•^ inhibition, followed by oven- and freeze-dried ginger. The high level of antioxidant activity in sun-dried extracts might be due to plants experiencing developmental changes and stress-induced responses due to sunlight [29]. Plants might sense the moisture loss and UVB-exposure as stress, and thus, synthesis of antioxidant compounds is induced to repair the damaged tissue and as a defense reaction from the injury [30]. In addition, a number of researchers have shown the strong links to sun exposure and vitamin D in reducing cancer risks and other oxidative effects, as reviewed by Holick [31]. In accordance with the previous literature, Chinese cabbage withered under sunlight for 2 days (UVB exposure) showed an increase in phenolic contents (i.e., hydroxycinnamic acids) in plants. Here, with regard to the biochemical processes in the plant, environmental stressors such as moisture loss and/or natural radiation from sunlight activated the protective secondary metabolite pathways [30] that result in the biosynthesis of phenolic antioxidants. This might explain why sun-drying might enhance the phytochemical content and potential bioactivity of ginger. 

### 3.3. Anti-Inflammatory Activity of Ginger Extracts

#### 3.3.1. Cell Viability

**Figure 1:**
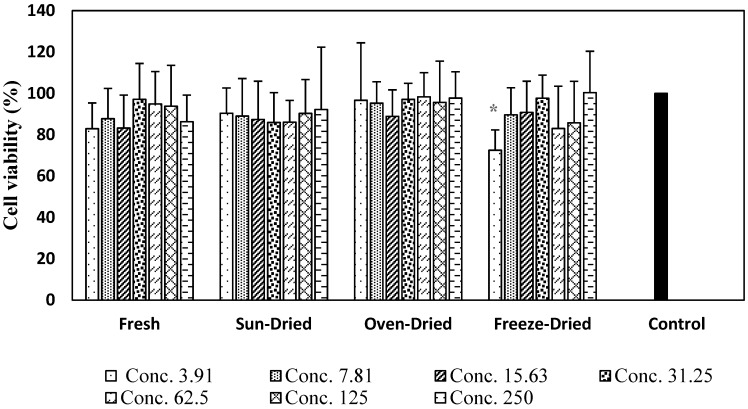
Effects of ginger extracts on viability of RAW 264.7 cells. The control (untreated cells) was taken as 100% viability. Values are mean ± SD from three independent experiments. * means statistically different at *p* < 0.05 between the different concentrations of the same extract when compared to the control.

Murine macrophage cell lines RAW 264.7 are the major cellular component and effector cells in response to inflammatory stimuli. The effects of fresh and dried ginger extracts in RAW 264.7 cells were measured by MTT assay. The cells were incubated in the presence of various concentrations of ginger extract (3.91–250 µg/mL). Figure 1 shows that the cell viability was not affected by treatment with up to 250 µg/mL. However, cell incubated with freeze-dried extracts at 3.91 µg/mL showed a significant decrease in cell viability compared to untreated control (*p* < 0.05). There were no significant differences in cell viability up to 250 µg/mL. Concentrations of 25, 50 and 100 µg/mL ginger extract (fresh and dried) were selected to determine the anti-inflammatory activity of ginger against NO inhibition.

#### 3.3.2. NO Production in LPS-Stimulated Cells

**Figure 2:**
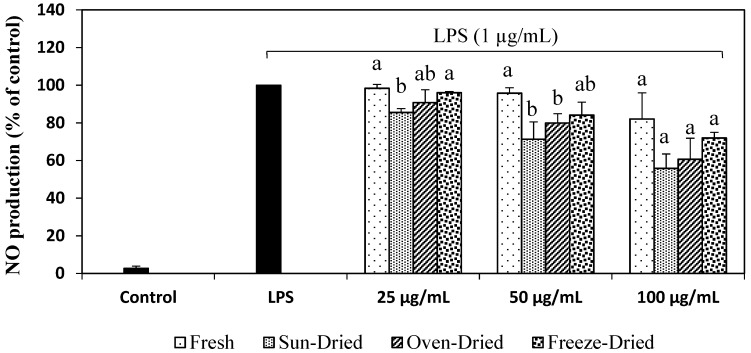
Effect of ginger extracts on the nitric oxide (NO) production in lipopolysaccharide (LPS)-stimulated RAW 264.7 macrophages. Values are mean ± SD from three independent experiments. Different letters indicate significant differences (*p* < 0.05) between the drying treatments within a same concentration.

Figure 2 shows the NO production in LPS-stimulated RAW 264.7 macrophages. In the present study, bacterial lipopolysaccharide (LPS) was applied to stimulate the RAW 264.7 cells, thus generating the NO as a pro-inflammation mediator. It was observed that the production of NO (100%) was markedly induced after cells were treated with 1 µg/mL LPS for 24 h, as compared to the unstimulated control (2.73%). In examining the ability of ginger extracts to suppress NO production in RAW 264.7 cells, cells were treated with concentrations of 25, 50 and 100 µg/mL of different extracts for all dried samples and the fresh sample. A concentration-dependent decrease in NO production was observed with ginger extracts in the presence of LPS (Figure 2). A decrease in NO production indicates that ginger extracts may help in treating chronic inflammatory diseases by reducing the NO levels. 

#### 3.3.3. NO-Inhibitory Activity of Ginger Extracts

**Figure 3:**
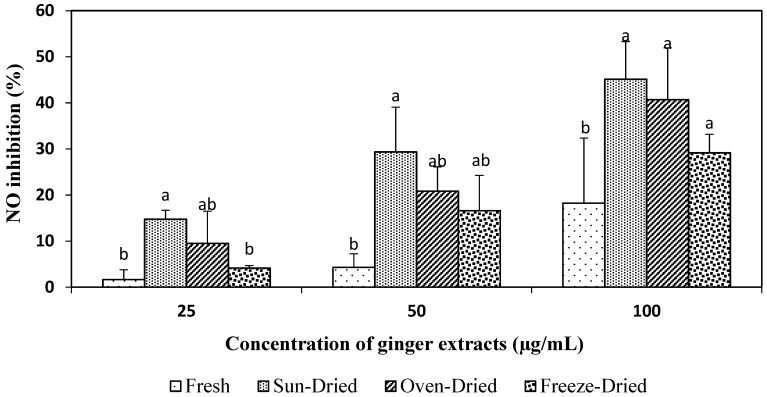
Effects of ginger extracts on the anti-inflammatory property (inhibition of NO). Values are mean ± SD from three independent experiments. Different letters indicate significant differences (*p* < 0.05) between drying treatments for each concentration.

Figure 3 shows that as the concentration increased from 25 to 50 and up to 100 µg/mL, the NO-inhibitory activity of dried extracts increased significantly to 14.75, 29.36, and 45.13% for sun-dried, 9.51, 20.84, and 40.71% for oven-dried, and 4.17, 16.60 and 29.16% for freeze-dried extracts, compared to 1.66, 4.31 and 18.23% for the fresh extracts. There is no significant difference (*p* > 0.05) in NO-inhibitory activity among the different drying treatments, except for the fresh ginger, especially at 100 µg/mL. 

Results of this study showed that all the ginger extracts for both fresh and dried ginger of *Halia Bara* had the ability to inhibit NO production in stimulated cells, and thus can be used as natural anti-inflammatory agents. Inhibition of nitric oxide production in vitro has been demonstrated for other ginger extracts and a considerable number of compounds isolated from them. A study done by Dugasani et al. [4] found that ginger extract at 6 µM caused 45–80% inhibition of NO production in LPS-stimulated RAW 264.7 cells. At concentrations of 10, 30 and 100 µg/mL, the NO-inhibitory activity of red ginger extracts increased in a dose-dependent manner from 5.3% to 74.6% [32]. Tsai et al. [7] and Li et al. [33] reported that ginger extract at 139 µg/mL and 7.79 µg/mL caused a 50% NO inhibition, which corresponded to their IC_50_ values. As a comparison, the present study found a 50% inhibition (IC_50_ values) of sun-dried extracts at 110 µg/mL, followed by oven-dried extracts at 122 µg/mL and freeze-dried at 162 µg/mL. Fresh extracts had the highest IC_50_ values with 241 µg/mL. 

Ginger extracts were found to be non-cytotoxic to RAW 264.7 cells (Figure 1); the result implies that ginger extracts inhibited nitric oxide without causing cell death. Dried *Halia Bara* demonstrated the best anti-inflammatory action with strong NO-inhibitory activity. This justifies the use of dried ginger in traditional systems of medicine for the treatment of various diseases related to oxidative stress and inflammation. 

In general, the drying process is a remarkable postharvest treatment for ginger as it provides enhanced antioxidant and anti-inflammatory properties through inhibition of NO production in LPS-stimulated RAW 264.7 cells. The drying process could cause the breakdown of several cellular constituents; nevertheless, it promotes the release of bound phenolic compounds from the food matrix [18] and induces the formation of new compounds with enhanced antioxidant properties [26]. In accordance to our study, the new compounds might be attributed to the formation of shogaols during drying, which have been reported to have more potent antioxidant properties and anti-inflammatory ability in dried ginger than in fresh ginger [4]. In addition, Roshanak et al. [18] reported that the low moisture content of dried samples causes the inactivation of destructive enzymes, and consequently, retains the maximum content of antioxidant compounds. The lower antioxidant activity observed in fresh ginger is possibly due to the presence of oxidative enzymes such as polyphenol oxidase (PPO), which cause the degradation of polyphenol compounds [18], and/or high moisture content, which prevents the complete release of essential volatile components of ginger [9]. 

The lower values of antioxidant properties and NO-inhibitory activity in oven-dried samples compared to the sun-dried could be attributed to the high temperature (60 °C) of oven heating. Oven heating has been reported to lead to the thermal degradation of phytochemical compounds and loss of antioxidant enzymes activity [26]. Pandey and Rizvi [34] also reported that high temperatures lead to oxidation reactions resulting in the deterioration of the quality of foods, particularly in browning and organoleptic properties. Although the freeze-dried extracts have the highest level of phenolic content (Table 1), they did not render to high antioxidant properties. It has been reported that antioxidant activity is dependent not only on its concentration but also on the structure and interactions between all antioxidants in the extracts. Consequently, samples with similar concentrations of phenolic compounds might differ significantly in their antioxidant properties [35]. 

## 4. Conclusions

The sun-drying process produced ginger extracts that showed remarkable results when compared with the oven- and freeze-drying methods. The sun-dried ginger extracts had significantly higher phenolic and flavonoids content and displayed the highest antioxidant activities of reductive ability of FRAP, the scavenging activity of ABTS^•+^ and DPPH^•^ inhibition. Although there no significant difference was found among the three drying methods of ginger extracts in terms of anti-inflammatory properties of NO-inhibitory activity, the sun-dried extracts had the lowest concentration required for 50% of NO inhibition (IC_50_ values) in LPS-stimulated RAW 264.7 cells. The sun exposure and vitamin D are plausible effects of the enhanced bioactivities of dried ginger. All dried ginger extracts showed significant improvement in their antioxidant and anti-inflammatory properties compared to fresh ginger extracts. 

## Figures and Tables

**Table 1:** Ginger *Halia Bara*’s extraction yield and its phenolic and flavonoid content.

Drying	Extract Yield (%)	Phenolic Content (mg GAE/g Dry Extract)	Flavonoid Content (mg RE/g Dry Extract)
Fresh	3.40 ± 0.80 ^b^	10.53 ± 0.21 ^d^	96.5 ± 4.08 ^c^
Sun-dried	9.87 ± 1.31 ^a^	18.94 ± 0.29 ^b^	584.8 ± 48.64 ^a^
Oven-dried	8.21 ± 2.79 ^a^	16.08 ± 0.52 ^c^	508.2 ± 38.80 ^ab^
Freeze-dried	9.50 ± 1.66 ^a^	20.07 ± 0.52 ^a^	473.2 ± 24.94 ^b^

Values are mean (*n* = 3) ± standard deviation and different superscripts letters within the same column are significantly different at *p* < 0.05.

**Table 2:** Ginger *Halia Bara*’s antioxidant activities of FRAP, ABTS^•+^ and DPPH^•^.

Drying	FRAP (mmol Fe (II)/g Dry Extract)	ABTS^•+^ (mmol TEAC/g Dry Extract)	DPPH IC_50_ µg/mL
Fresh	1021 ± 29.7 ^d^	829 ± 44.0 ^c^	65.82 ± 6.83 ^a^
Sun-dried	4033 ± 29.9 ^a^	1712 ± 1.7 ^a^	14.69 ± 2.34 ^c^
Oven-dried	3584 ± 61.1 ^b^	1428 ± 51.8 ^b^	27.97 ± 1.92 ^b^
Freeze-dried	3219 ± 72.5 ^c^	1336 ± 72.8 ^b^	28.59 ± 0.83 ^b^

Values are mean (*n* = 3) ± standard deviation and different superscripts letters within the same column are significantly different at *p* < 0.05. FRAP = Ferric-Reducing Antioxidant Power, ABTS^•+^ = radical cation 2,2′-azino-bis(3-ethylbenzothiazoline-6-sulfonic acid), and DPPH^•^ = radical 1,1-diphenyl-2-picryl-hydrazyl.
